# Comparative Assessment of Antibiotic Residues Using Liquid Chromatography Coupled with Tandem Mass Spectrometry (LC-MS/MS) and a Rapid Screening Test in Raw Milk Collected from the North-Central Algerian Dairies

**DOI:** 10.3390/toxics10010019

**Published:** 2022-01-05

**Authors:** Fawzi Rostane Meklati, Anthi Panara, Ahmed Hadef, Amel Meribai, Meriem H. Ben-Mahdi, Marilena E. Dasenaki, Nikolaos S. Thomaidis

**Affiliations:** 1 Centre de Recherche Scientifique et Technique en Analyses Physico-Chimiques CRAPC, BP 384 Bou-Ismail, Tipaza 42004, Algeria; meklati4@gmail.com; 2 Laboratoire de Recherche «Santé et Productions Animales», Ecole Nationale Supérieure Vétérinaire ENSV, Rabie Bouchama, Oued-Smar, Algiers 16000, Algeria; mh.benmahdi@ensv.dz; 3 Laboratory of Analytical Chemistry, Department of Chemistry, National and Kapodistrian University of Athens, Panepistimiopolis Zographou, 15771 Athens, Greece; panaranthi@chem.uoa.gr; 4 Department of Veterinary Sciences, Faculty of Nature and Life Sciences, Chadli Bendjedid University of El Taref, PB 73, El-Taref 36000, Algeria; hadef_vet@yahoo.fr; 5 Laboratory of Development and Control of Hospital Pharmaceutical Preparations, Faculty of Medicine, Badji Mokhtar University of Annaba, Annaba 23000, Algeria; 6 Laboratoire de Recherche en Technologie Alimentaire et Nutrition Humaine, Ecole Nationale Supérieure Agronomique, Algiers 16004, Algeria; a.meribai@gmail.com; 7 Laboratory of Food Chemistry, Department of Chemistry, National and Kapodistrian University of Athens, Panepistimiopolis Zographou, 15771 Athens, Greece

**Keywords:** antibiotics, contaminant residues, LC-MS/MS, MRL, rapid screening test, raw milk

## Abstract

Antibiotic residues in milk are a major health threat for the consumer and a hazard to the dairy industry, causing significant economic losses. This study aims to assess the presence of antibiotic residues in raw milk comparatively by a rapid screening test (BetaStar^®^ Combo) and Liquid Chromatography coupled with Tandem Mass Spectrometry (LC-MS/MS). A total of 445 samples were collected from 3 dairy companies of north-central Algeria (Algiers, Blida, Boumerdes), and they were rapidly screened for β-lactams and tetracyclines; 52 samples, comprising 34 positive tanker-truck milk and 18 negative bulk-tank milk were tested by LC-MS/MS, which revealed 90.4% were contaminated (*n* = 47) and 55.3% exceeded the Maximum Residue Limit (MRL). The β-lactams as parent compounds and their metabolites were the most frequently detected with maximum value for cloxacillin (1231 µg/kg) and penicillin G (2062 µg/kg). Under field condition, the false-positive results, particularly for tetracyclines, seems to be related to milk samples displaying extreme acidity values (≥19°D) or fat-level fluctuations (2.7 g/100 mL and 5.6–6.2 g/100 mL). Despite a relatively low prevalence (7.64%) of residues using the rapid test, the detection by LC-MS/MS of flumequine (52 µg/kg), cefaclor (maximum 220 µg/kg) and metabolites of β-lactams at high levels should lead to reflections on the control of their human and environmental toxicological effects.

## 1. Introduction

In Algeria, the dairy sector is one of the main cornerstones of agricultural development, but it has so far experienced a delay in its development due to various technical and economic difficulties [1]. As in many developing countries, the successive restructuring programs of the dairy sector established in recent decades have mostly focused on the quantitative aspect leading to increase milk production; performance remains very insufficient to cover the requirements of a growing population. Nevertheless, the quality of the milk produced should meet public health guidelines. Thus, quality control of this food of animal origin, including antibiotic-residue monitoring, is an important concept that is absolutely necessary to ensure dairy safety for the consumer [2,3].

In contrast to the European context, the screening for antimicrobial residues in food products of animal origin has received limited interest from public authorities in developing countries [4]. To overcome these shortcomings by providing a healthy and economically profitable product for domestic and international market, a program (DZ/13/ENP/HE/17) of partnership between Algeria and the European Union (EU) was created in 2013. In the same framework, a European twinning project carried out by a France–Italy consortium for the benefit of Algerian Veterinary Services was launched in April 2014. The project resulted in the strengthening of veterinary controls to bring them up to European standards involving PASCRA (Plan Algérien de Surveillance des Contaminants dans les Résidus Alimentaires), the Algerian plan for the surveillance of contaminants and residues in food [5].

Contamination of foodstuffs with antibiotic residues, both as parent compounds and their metabolites, is liable to produce a multitude of effects on the consumer that can be highly detrimental to human health. Some studies have reported cases of antibiotic resistance in bacterial strains isolated from animal production [6,7], as well as allergic reactions, toxicity, teratogenicity and carcinogenicity [8,9]. For agribusiness and, more specifically, the dairy processor, their presence had a technological impact with severe financial consequences through the loss of the raw material by delaying or totally inhibiting the necessary fermentation processes in cheese and yogurt manufacturing [10,11]. Nevertheless, up to now, unfortunately, no national official data from public authorities are available on the prevalence of veterinary antimicrobial residues in food matrices of animal origin or on their level of use by practitioners on livestock farms. In addition, Algeria recently introduced a regulatory text [12] setting lists and Maximum Residue Limits (MRLs) of residues of veterinary drugs or pharmacologically active substances tolerated in foodstuffs of animal origin in line with current international regulations [13].

Nevertheless, it is important to underline that even before the adoption of these latest regulations, most of the licensed Algerian dairy industries had adopted some methods of screening for antimicrobial residues in milk, with a particular emphasis on screening for β-lactams and tetracyclines residues. These methodologies were selected on the basis of technical criteria, avoiding inhibition of fermentative processes during manufacture, and practical reasons such as the fast-result reading, sensitivity to MRLs and test cost. Both tetracyclines and β-lactams are mainly detected using the BetaStar^®^ Combo test kit [14,15].

In the absence of official authorities’ data, only a few field works have been conducted in some Algerian regions to detect antibiotic residues in milk using microbial inhibitor tests [2,16,17]. However, these qualitative methods are not sensitive enough in some cases and could generate false-positive and false-negative results [18,19]. Indeed, studies on antimicrobial residues should be more focused on the thorough identification and quantification of the target molecules using modern confirmation methods such as Liquid Chromatography coupled with Tandem Mass Spectrometry (LC-MS/MS) [20,21,22]. LC-MS/MS and other advanced analytical techniques have been involved in the validation of qualitative screening tests such as the BetaStar^®^ Combo test, which has already been extensively presented in the literature on cow [23], ewe [24,25] and goat milk [26].

Far from the objective of revalidating this rapid-screening test, the present work aims first of all to evaluate under field conditions the quality of milk with regard to the presence of antimicrobial residues belonging to β-lactams and tetracyclines. This appraisal was performed on milk sampled at delivery and before processing in three dairy industries whose products are widely available on the market in the metropolitan and peri-urban regions of north-central Algeria (Algiers, Blida, Boumerdes). Secondly, the results of BetaStar^®^ Combo were compared with those obtained by the LC-MS/MS method applied on selected samples of raw-milk tanker trucks and bulk-tank milk of companies in order to verify the accuracy of detecting antimicrobial residues that reach the β-lactams and tetracyclines’ MRLs threshold. As a third purpose, the identification and quantification of sulfonamides, quinolones and macrolides were also investigated in order to provide a wider perception regarding the presence of antibiotic residues in milk samples.

## 2. Materials and Methods

### 2.1. Milk Sampling

**Figure 1:**
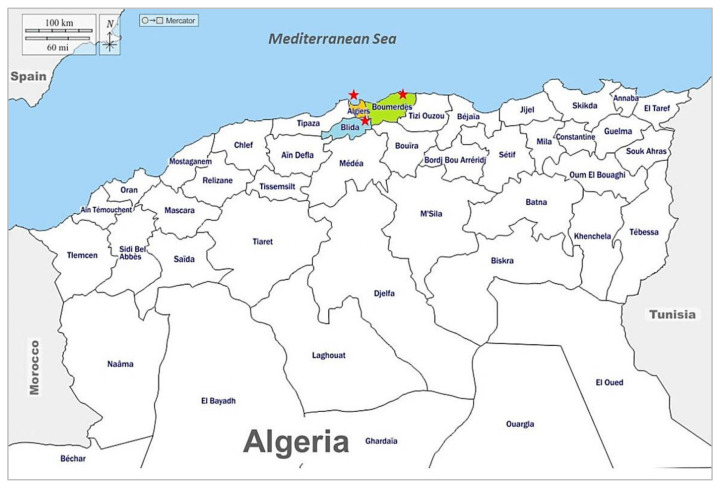
Geographic localization of the three investigated dairy companies.

The study was conducted in north-central Algeria comprising 10 provinces where 38.7% of the dairy industries are affiliated with the public institution ONIL, the National Interprofessional Milk Office [1]. Based on the geographical distribution of dairy industries and the particular attention given to the milk hygienic quality in this area, we selected three dairy companies (Figure 1): Colaital Algiers (company A), Laiterie-Fromagerie de Boudouaou (company B) and Tlemsani Agro (company C), located respectively in each of the three provinces (Algiers latitude 36°43′36.6″ N, longitude 3°02′57.3″ E; Boumerdes latitude 36°43′39.4″ N, longitude 3°23′51.8″ E; Blida latitude 36°29′31.8″ N, longitude 2°50′28.6″ E).

In all surveyed industries, raw milk is transported by a tanker-truck network, involving 30 collectors carrying out daily collection from 301 dairy farms (186 in Algiers, 77 in Boumerdes and 38 in Blida). Colaital (company A) has a large production capacity, estimated at 300,000 L per day, intended to market Conditioned Pasteurized Milk (CPM) in plastic packages and derivative products, mainly butter and yogurt. Milk was delivered from farms in 10 districts (Birkhadem, Birtouta, Cheraga, Ouled Chebel, Souidania, Zeralda, Ain Taya, Dar El Beïda, H’raoua, and Reghaïa, Rouïba). Laiterie-Fromagerie de Boudouaou (company B), for its part, produces CPM at a rate of 400,000 L per day, and processed and EDAM cheese. Company B’s milk is delivered from farms located in 17 districts (Afir, Ben choud, Boudouaou, Boumerdes, Corso, Dellys, Hamadi, Khemis el Khechna, Legata, Ouled Heddadj, Ouled Hellal, Ouled Moussa, Reghaïa, Rouïba, Thenia, Tijelabine, and Zemmouri). Finally, the Tlemsani Agro cheese industry (company C) produces exclusively a Camembert type cheese with an annual production valued at 416 tons. The milk is mainly received from farms distributed in 9 districts (Birtouta, Blida, Boufarik, Ahmeur El Ain, Attatba, Berbessa, Bourkika, Kolea, Sidi-Rached). The products of all three companies under study are marketed in the main sales outlets and supermarkets of the capital, as well as in the districts of the north-central region of the country.

A total of 445 raw milk samples (186 at company A, 140 at company B, and 119 at company C) were collected from February to April 2016 and analyzed the same year. A volume of 0.5 L was sampled directly from the milk tanker trucks upon delivery and from the bulk-tank milk for immediate analysis or were stored at −20 °C for further processing.

### 2.2. Titratable Acidity and Milk-Fat Level Measurements

For all the milk samples, titratable acidity and fat level (FL) were measured according to AFNOR standards [27,28].

### 2.3. Procedure Applied for the Screening of Antibiotic Residues in Milk

Antibiotic residues were monitored upon milk delivery to the quality-control laboratories of all the dairy companies. In a first step, a screening of samples was carried out using the BetaStar^®^ Combo test (Neogen Corporation, Lansing, MI, USA) to detect antibiotic residues belonging to the β-lactams and tetracyclines’ families. In a second step, all BetaStar^®^ Combo positive samples that could suggest the presence of antimicrobial residues, along with some samples randomly collected from the bulk tanks of each dairy industry intended for processing into by-products, were immediately placed in polypropylene containers to be frozen at −20 °C for 24 h before being freeze-dried for 96 h with the Alpha 2–4 LSCplus CHRIST freeze-dryer (Martin Christ Gefriertrocknungsanlagen GmbH, Osterode am Harz, Germany). The temperature of the condenser was −40 °C at a vacuum pressure of 0.28 mbar. This step was performed to facilitate preservation of the milk samples. The freeze-dried milk powders obtained were stored hermetically at −20 °C until their analysis at the Laboratory of Analytical Chemistry of the National and Kapodistrian University of Athens, by LC-MS/MS, in order to confirm their positivity and also to identify and quantify the compounds, parent and metabolites, contaminating milk samples. This storage approach does not affect the stability of antibiotics and their residues in milk samples, as investigated by Chen et al. [29] for cloxaxillin and Gbylik-Sikorska et al. [30] for fluoroquinolones.

### 2.4. BetaStar^®^ Combo Screening Method

The regulatory framework regarding performance and validation criteria for analytical methods to screen residues of antimicrobial drugs in foodstuffs is not yet established in Algeria, conversely to the European Union. Nevertheless, the BetaStar^®^ Combo monitoring test performed in this study was in compliance with the requirements for validation criteria of analytical methods in accordance with European Decision 2002/657/EC [31]. According to the manufacturer (Neogen Corporation, Lansing, MI, USA), the detection limit reveals residues of both β-lactams and tetracyclines at levels that may be inferior for some compounds regarding the Maximum Residue Limits (MRLs), as defined by the European Regulatory Commission 2010/37/EC [32], with an exception for cefalexin and desfuroylceftiofur (Table 1). The rapid test (5 min) is based on an immunochromatographic medium that uses a specific receptor attached to gold particles. A volume of 0.2 mL of each milk sample to be tested was deposited in a vial containing the active reagent and then was incubated at 47.5 ± 1 °C for 2 min. During this first incubation step, the antibiotics β-lactams and tetracyclines, when present in the milk sample, bind to the receptors. A dipstick with three capture lines provided by the manufacturer was placed in the vial. In this second step, incubation was carried out at the initial temperature (47.5 ± 1 °C) for 3 min. At the end, the dipstick was removed from the vial and the result was immediately interpreted by comparing the coloration intensity of the upper line (with reference to β-lactams) and of the bottom line (with reference to tetracyclines) with that of the middle (control line) used as a control. The visual interpretation of the result was performed according to the manufacturer’s recommendations. An upper line with a weak or even no coloration intensity compared to the control line would indicate the presence of residues of antibiotics belonging to β-lactams. In the same way, the bottom line was interpreted, revealing residues of the tetracyclines family antibiotics. For both families, when the coloration of the two lines was equal to or more intense than the reference band, the test was considered negative, with no antibiotics in the milk sample evaluated.

### 2.5. Quantitative LC-MS/MS Methodology

#### 2.5.1. Chemicals and Reagents

All veterinary standards used were of high purity grade (>90%). Amoxicillin, cefaclor, cefadroxil, cloxacillin and oxacillin sodium salt monohydrate, ampicillin trihydrate, penicillin V potassium salt, dicloxacillin sodium salt hydrate, cefazolin sodium salt, cefalexin, cefquinome sulfate, cefalonium hydrate, ceftiofur, chlortetracycline, doxycycline, oxytetracycline, tetracycline, minocycline, sulfadiazine, sulfathiazole, sulfamerazine, sulfadimidine, sulfamethoxypyridazine, sulfamonomethoxine, sulfachloropyridazine, sulfadimethoxine, sulfamethizole, sulfamethoxazole, sulfisoxazole, sulfaguanidine, sulfapyridine, sulfamoxole, sulfaquinoxaline and trimethoprim were purchased from Sigma-Aldrich (Steinheim, Germany). Cefapirin sodium was purchased from Santa Cruz Biotech (Heidelberg, Germany), while penicillin G sodium salt and cefoperazone sodium salt were purchased from Alfa Aesar (Karlsruhe, Germany). Sulfadoxine and sulfaclozine were donated by the National Laboratory of Residue Analysis of Food of Animal Origin of the Hellenic Ministry of Rural Development and Food. Quinolones used were ciprofloxacin, danofloxacin, difloxacin, enrofloxacin, flumequine, marbofloxacin, norfloxacin, olfloxacin, oxolinic acid, sarafloxacin, while macrolides employed were azithromycin, clarithromycin, erithromycin, tiamulin, tilmicosin, tylosin.

Acetonitrile and methanol LC–MS grade were purchased from Merck (Darmstadt, Germany), while formic acid 99% and ammonium formate were supplied from Fisher Chemical (Geel, Belgium). Sodium hydroxide monohydrate (NaOH) for trace analysis ≥99.9995% was purchased from Fluka (Buchs, Switzerland); n-Hexane (analysis grade, EMSURE^®^ ACS) was purchased from Merck KGaA (Darmstadt, Germany). Water was provided by a Milli-Q system (Millipore Direct-Q UV, Bedford, MA, USA). Potassium phosphate dibasic anhydrous of analytical grade was purchased from Carlo Erba (Val de Reuil, France). The ethylenediaminetetraacetic acid disodium salt (EDTA) was of analytical grade and was obtained from Panreac (Barcelona, Spain). Syringe filters Regenerated Cellulose (RC), 0.22 μm pore size were supplied from Phenomenex (Torrance, CA, USA). Solid phase extraction (SPE) Strata-X 33 µm polymeric reversed phase, 200 mg/6 mL cartridges were purchased from Phenomenex (Torrance, CA, USA).

#### 2.5.2. Preparation of Standards

Independent preparation of individual standard substances was performed at a final concentration of 1 mg/mL. Sulfonamide, tetracycline, quinolone and macrolide standards were dissolved in methanol, while β-lactams and cefalosporines were dissolved in water (Milli-Q water). To enhance their solubility, 100 µL of formic acid was added to the quinolone standards. Stock solutions were stored in brown glass at −20 °C, except for β-lactams and cefalosporines, which were stored at 4 °C. Two mixtures of working standard solutions at a concentration of 1 µg/mL each were prepared independently, one containing β-lactams and cefalosporines in water, while the other solution contained a mixture of tetracyclines, sulfonamides, macrolides and quinolones in methanol. The mixtures were stored under refrigerated conditions at 4 °C for a period not exceeding 1 week.

#### 2.5.3. Milk Samples Preparation

For the determination of β-lactams, an in-house method was applied based on the SPE-protocol developed by Hou et al. [33]. Specifically, 1 gram of homogenized freeze-dried milk sample was weighed and inserted into a 50 mL polypropylene tube, and then a volume of 45 mL of phosphate buffer solution (PBS) was added. This PBS buffer was previously prepared by dissolving 2.176 g of potassium phosphate dibasic anhydrous in 250 mL of water, while adjusting the pH to 8.5 using 0.1 M solution of NaOH. Adjustment of the PBS buffer solution pH to this value is essential for the recovery of penicillins and cefalosporines from milk. After horizontal stirring (15 min) until completely dissolved, samples were placed into an ultrasonic bath at 40 °C for 15 min. A volume of 1.5 mL of acetonitrile was added followed by vortex agitation for milk deproteinization. Centrifugation at 4000 rpm for 10 min was performed for defatting; the supernatant was pipetted and recovered in a new tube to be stored for 12 h at −20 °C to remove the residues of lipid and protein materials. The blank and spiked samples (50 µL and 150 µL added from the working standard mix solutions of penicillins and cefalosporines at 1 µg/mL) were prepared in the same conditions. All samples were subsequently cleaned up by SPE (Strata X cartridges) to remove interferences from the matrix and promote the collection of stable extracts. The cartridges were preconditioned with 10 mL methanol and 5 mL PBS buffer solution (0.05 M; pH 8.5), and then the sample was loaded onto the cartridge first without flow and then with a slight flow (2 mL/min) using vacuum. In order to remove interferences, a washing step was performed with 3 mL PBS and 2 mL water. The SPE cartridges were then dried for 5 min under vacuum, and the target analytes were eluted using 5 mL acetonitrile. The samples were evaporated to dryness at 40 °C and reconstituted with 250 µL of ammonium formate 5 mM: MeOH (95:5, *v*/*v*). Validation data for this in-house method are provided in Table S1. For the determination of tetracyclines, macrolides, sulfonamides and quinolones, extraction was carried out according to the Dasenaki and Thomaidis [34] method, by weighing 1 g of each freeze-dried milk and blank samples. Two fortified spiked samples were additionally prepared from the working standard solution by adding 150 µL and 300 µL, respectively. Milk proteins were removed by successive addition followed by vortex stirring (30 s) of 8 mL 0.1% EDTA in water with 0.1% formic acid, 3 mL acetonitrile and 3 mL methanol. The samples were then placed in an ultrasonic bath at 60 °C for 20 min and centrifuged at 4000 rpm for 10 min. The supernatant was moved to a new tube at −20 °C overnight to precipitate the remaining protein and then removed after centrifugation (4000 rpm). The supernatant was transferred once again to a new tube, followed by the addition of 5 mL of hexane to remove the milk fat. After vortex agitation and centrifugation (5 min), the hexane layer was discarded and the tubes were evaporated to dryness at 40 °C and reconstituted in 1 mL of methanol/aqueous solution of formic acid, 0.05% (25:75 *v*/*v*).

#### 2.5.4. LC-MS/MS Analysis

After reconstitution, each sample was filtered through 0.22 μm Regenerated Cellulose (RC) syringe filters, and then the extract was placed in an amber vial and a volume of 10 µL was injected in the LC-MS/MS system. The LC-MS/MS used for antibiotic determination was a Thermo Fisher Scientific (San Jose, CA, USA) Accela UHPLC Thermo system connected to the Access Quantum Thermo Scientific TSQ triple quadrupole instrument with autosampler. The compound ionization was performed using Electrospray Ionization (ESI) in positive ionization mode and the mass spectrometer parameters were: Spray Voltage 4000 V, Seath Gas 25 psi, Auxiliary Gas 10 a.u., Capillary Temperature 300 °C. Two different chromatographic methods were used for the determination of β-lactams and all other analytes. An Atlantis T3 C18 (100 mm × 2.1 mm, 3 µm, Waters) chromatographic column, protected by a pre-column, was used for antimicrobial residues’ separation at a constant flow rate of 100 µL min^−1^. For the determination of β-lactams, two mobile phases were prepared consisting of water with 0.1% formic acid (solvent A) and methanol with 0.1% formic acid (95:5, *v*/*v*) (solvent B). For the investigation of the other antibiotic families, the first mobile phase consisted of water with 0.1% formic acid (solvent A) and methanol (solvent B). The limit of detection (LOD) values under our LC-MS/MS conditions were reported in Table 1. The gradient elution programs used are presented in the ESM (Table S2). The quantification ion is highlighted in bold in Table S3, which contains the multiple reaction monitoring (MRM) parameters, retention times and ion ratio for each target compound. Data acquisition and instrument control were performed using Xcalibur software, Version 2.3 (Thermo Fisher). The concentrations of antibiotic compounds in each confirmed positive sample were calculated according to the following equation:

Concentration of sample (µg/kg) = (Peak area of sample × concentration of spiked blank sample)/(Peak area of spiked blank sample).

Along with the determination of β-lactams as parent compounds, the determination of 5 metabolites was also performed. Penicillin G, penicillin V, cloxacillin, dicloxacillin, and oxacillin were also monitored as their [(M + H_2_ − CO) + H] + degradation products as they are easily subjected to a β-lactam ring-opening that leads to the formation of these products. Amoxicillin and ampicillin, due to their structure (−NH_2_ at the R ring), are stable even in very acidic conditions and do not form such degradation products [35,36]. The quantification of the metabolites was performed using the corresponding parent compound (semi-quantification methodology).

### 2.6. Statistical Analysis

The data were analyzed using IBM^®^ SPSS^®^ Statistics 26 software. Descriptive statistics were conducted to illustrate the results of the screening of milk samples by BetaStar^®^ Combo test as well as by LC-MS/MS, and their characteristics were compared across companies (categorical variables) using the “z” test for multiple comparison. The Chi-squared test was used to assess the relationship between the positivity of samples to the screening tests and the sampling origin (company). The accuracy of the BetaStar^®^ Combo test compared to LC-MS/MS was evaluated using the receiver operating characteristic curve (ROC curve). The Student’s *t*-test was performed to compare the observed means of Fat Level (FL) and Acidity (dependent variables) with the reference values and to evaluate their means differences according to the screening tests’ results (grouping variable). Variance analysis (ANOVA) was conducted to assess the differences among means of physicochemical parameters of milk samples across companies (independent variable) using Tukey’s HSD post hoc test for multiple comparison. In addition, Pearson’s correlation was applied to quantify the relationship between the studied variables (FL, acidity, samples origin, and the screening positivity). For all tests, in order to reach a conclusion, a critical significance level of 0.05 (5%) was assumed.

## 3. Results and Discussion

### 3.1. Detection of Antibiotic Residues by BetaStar^®^ Combo and LC-MS/MS

#### 3.1.1. Antibiotic Residue Screening by BetaStar^®^ Combo

The screening for antibiotic residues applied to 445 samples collected from tanker-truck and bulk-tank milk from each dairy company by BetaStar^®^ Combo rapid test (Table 2) revealed 34 positive samples (7.64%) and 411 negative ones (92.4%). This prevalence is higher than that recorded by Debeche et al. [14] (3.25%) from 10,153 bulk-tank milk samples screened by BetaStar^®^ Combo in the region of Msila (Algeria) and that of Bilandžić et al. [37] in Croatia (3% of 1259 samples) after a screening by the Delvotest SP-NT (DSM Food Specialties Ingredients, The Netherlands). In contrast, BetaStar^®^ Combo permitted Pogurschi et al. [38] to record a higher level (31.4% of 210 samples) of antibiotic residue contamination in milk collected in the Bucharest metropolitan area (Romania) than our findings.

Compared to the results of other studies including screening methods, the values obtained were slightly lower than those reported by Ben-Mahdi and Ouslimani [39] in the central area near Algiers (9.87% of 760 samples). Markedly higher rates were also reported in the central-northern area of Algeria by Titouche et al. [40] (46.8% of 171 samples) and by Mimoune et al. [17] (18.1% of 160 samples) using microbiological methods, as well as by Aggad et al. [16] and Layada et al. [41] in western and eastern Algeria (respectively, 29.0% of 83 samples and 65.5% of 194 samples), via the Delvotest^®^ SP-NT.

Taking into account the geographical distribution of the screening results by sampling province, low contamination with antimicrobial residues in the Algiers and Boumerdes regions (4.84% and 4.29%, respectively) was reported. These values are significantly smaller than those reported by Mimoune et al. [17] using individual cow’s milk from farms in these 2 wilayas (20.8% and 17.0%, respectively). Compared to the latter two provinces, the Blida area (company C) was the most affected by the presence of antimicrobial residues in milk with a 4 times (16.0%) higher prevalence (Table 2) that is slightly above that established by Baazize-Ammi et al. [15] in the same region (12.0%) using the Delvotest^®^ SP method. Authors also noticed lower milk contamination rates with BetaStar^®^ Combo compared to Delvotest^®^ SP-NT, which has an extended sensitivity to other antibiotic groups (sulfonamides, macrolides, aminoglycosides, trimethoprim and dapsone) besides β-lactams and tetracyclines. Hamiroune et al. [2] reported 28.8% of milk samples in the same region (Blida) to contain bacterial inhibitors by Delvotest^®^ SP-NT.

Referring to the antibiotic family and for all the companies studied, the screening test revealed a predominance of β-lactams (91.2%) over tetracyclines (20.6%) that were only present at the Blida cheese factory, either alone (36.8%) or simultaneously with β-lactams (21.1%). This β-lactams family preponderance is congruent with the results of Baazize-Ammi et al. [15] obtained following a screening using BetaStar^®^ Combo. Conversely, Pogurschi et al. [38] in Romania noticed via the same test a predominance of tetracyclines (81.8%) over β-lactams (18.2%). At the African continental scale, tetracyclines seemed to be the most detected antibiotic family (41.0% of all contaminants), followed by β-lactams (18.0%) as reviewed by Darwish et al. [8].

#### 3.1.2. Antimicrobial Residue Detection by LC-MS/MS

The LC-MS/MS analysis of 52 milk samples (34 positive tanker-truck and 18 negative bulk-tank samples) of dairy companies revealed the presence of antimicrobial residues in 47 samples (90.4%), of which 26 (55.3%) were positives exceeding the MRL (Table 2). This prevalence of antimicrobial residues detected by LC-MS/MS was significantly higher than that using the BetaStar^®^ Combo rapid-screening test (65.4%). This difference could be explained in part by the LC-MS/MS capability to identify compounds that cannot be detected by the BetaStar^®^ Combo. In addition, as presented in Table 1, LODs achieved for β-lactams with LC-MS/MS were significantly lower than those obtained using BetaStar^®^ Combo (more than 4 times lower). The low LODs obtained enabled their identification in very low concentrations. Many studies conclude that powerful techniques using liquid chromatography provide higher precision and accuracy than rapid-screening tests [42,43].

The LC-MS/MS analysis resulted in the identification of 161 compounds from 52 assessed samples (Table 3) belonging to 5 antibiotic families (β-lactams, tetracyclines, fluoroquinolones, sulfonamides and diaminopyrimidines).

**Figure 2:**
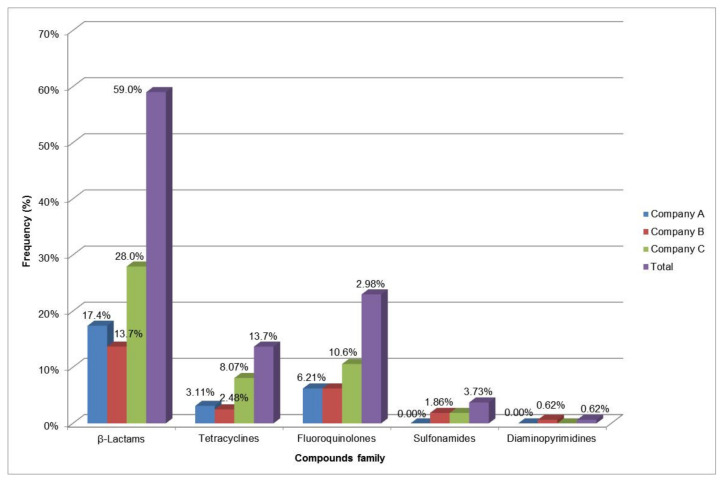
Rank order of antibiotic groups evaluated by company.

For all surveyed companies, β-lactams (penicillin G, cloxacillin, dicloxacillin, oxacillin), as the parent compound and their metabolites, were the most frequently detected residues by LC-MS/MS (59.0% of 161 compounds), coming in agreement with the BetaStar^®^ Combo screening results for this antibiotic family (Table 3 and Figure 2). These results also prove the dominant position held by β-lactams among veterinary drugs used in Algeria, previously discussed by Ben-Mahdi and Ouslimani [39]. The findings from the surveys reviewed by Sachi et al. [44] on the occurrence of antibiotic residues in the milk worldwide highlighted the first place held by β-lactams (36.5%). According to geographical origin, the prevalence (28.0%) of this antibiotic family is markedly higher in company C than in the other two, companies A and B.

**Figure 3:**
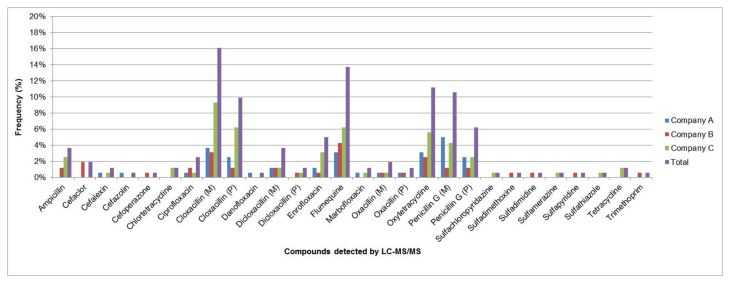
Distribution and frequency of antibiotic residues in companies.

Among the β-lactams, cloxacillin and penicillin G (as the parent compound and their metabolites) were the most commonly identified compounds (up to 50.0% of 52 samples), with maximum concentrations of 1231 µg/kg and 2062 µg/kg exceeding the MRL for each compound by 41 to 515 times, respectively. Maximum concentrations of cloxacillin and penicillin G were higher than those reported on milk collected in Kosovo during 2009 and 2010 as the most frequently detected molecules (542–1973 µg/kg, respectively) [45]. Moreover, these two antibiotics are widely used in Algeria for the treatment of bovine mastitis, which is considered the dominant pathology in dairy farms causing significant economic losses [46]. Within compounds, cloxacillin as the parent compound and its metabolite were the most frequently evaluated molecules. They were mainly detected in samples from company C (Figure 3).

Commonly used worldwide in animal therapeutics, maximum penicillin G concentrations in milk were almost 6-fold higher than the value reported by Khanal et al. [47] in Nepal (353 µg/kg). However, they were 3-fold lower than those recorded in Italy by Ghidini et al. (6058.25 µg/kg) [18].

Very elevated maximal contents were also noted for dicloxacillin as the parent compound and its metabolite (413–893 µg/kg) and ampicillin (309 µg/kg and a prevalence of 11.5%), exceeding the MRL by 13 up to 77 times. In 2 other milk samples, the MRL was exceeded for oxacillin (36 µg/kg) and cefalexin (111 µg/kg), while in 1 other sample a cefazolin concentration at the MRL threshold (50 µg/kg) was recorded. The conspicuous presence of β-lactam residues could represent a human health hazard by inducing allergic reactions [48]. In addition, their presence as parent compounds and their metabolites could delay or completely prevent the fermenting processes [49].

Fluoroquinolones were the second most frequently identified group (23.0%) after β-lactams (Figure 3), of which almost half (10.6%) were from company C. Over the past few years, a steady increase in quinolones use had been noticed in Africa [8]. In the present study, this antibiotic family was mainly accounted by flumequine (42.3% prevalence from 52 samples), even though only one sample (52 µg/kg) from company A exceeded the MRL (50 µg/kg). Enrofloxacin was present in 15.4% of the samples evaluated, of which only one sample was at the MRL threshold (100 µg/kg), followed by ciprofloxacin (7.69%). For the latter compound, all detected concentrations were below the MRL (100 µg/kg), which is in agreement with the results of Tasci et al. in Turkey, in which ciprofloxacin was identified in only 1 milk sample out of 68 with a concentration of 0.012 μg/mg [50].

Tetracyclines, which were screened (BetaStar^®^ Combo) and confirmed (LC-MS/MS), were the third most detected (13.7%) antibiotic family (Figure 3). Among that group, oxytetracycline was the most commonly identified compound (34.6%), with two samples from company C exceeding the MRL of 100 µg/kg (179 and 660 µg/kg). These results are in line with those of Al-Mazeedi et al. [51], who reported a dominance of oxytetracycline (80% of the family) although they recorded a higher prevalence (37.0%) of tetracycline residues in raw milk in Kuwait with a maximum value of 350 µg/kg. In addition, we identified 1 sample showing a huge concentration for tetracycline (2291 µg/kg). The same phenomenon was also reported in Croatia by Bilandžić et al. [37]. This could be in relation to the fact that tetracyclines, enrofloxacin and ciprofloxacin are generally used for prevention and treatment of livestock diseases and, consequently, they could be a potential risk to the consumer at the end of the food chain if not detected [52].

Sulfonamides and trimethoprim displayed a prevalence of 1.92% for each (1 sample out of 52) with maximum concentrations of 58.0 (sulfadimidine) and 16.0 μg/kg, respectively, both under the MRL threshold according to the European regulation [32]. Recorded on a single sample from Company B, the concentration (58.0 μg/kg) of sulfadimidine was, however, exceeding the MRL (25 µg/kg) established by the Algerian regulation [12]. These results are consistent with those reported by Han et al. [53] in Hubei (China), who assessed sulfonamides and trimethoprim in milk at maximum concentrations of 9.3 and 20.7 μg/kg, respectively. However, the results are weaker than those obtained by Orwa et al. [54] in Kenya, in which 60% of positive samples were accounted for sulfonamide residues.

An unexpected presence of cefaclor was recorded in 3 samples (prevalence of 5.77%) of milk from company B (maximum concentration of 220 µg/kg), even though this substance is not intended to be used in milk-producing animals for human consumption and there are no MRLs established for this second-generation cephalosporin. This antibiotic, restricted to human use and prescribed in many countries, has been detected at high concentrations in sewage-treatment-plant wastewater [55,56] and could easily, if some precautions are not taken in the event of release into the environment, end up in animal productions at the end of the food chain. In addition, some pharmaceutical industries specializing in the manufacture of medicines, including antibiotics for human use, are located in industrial areas through the wilaya of Boumerdes. It is important to highlight that according to the manufacturer’s guidelines, the BetaStar^®^ Combo has no scope for fluoroquinolones, sulfonamide, trimethoprim and cefaclor, and therefore cannot detect these compounds in contaminated milk samples. If these molecules are not detected by other methods such as LC-MS/MS, they will inevitably end up in milk, with serious health risks for the consumer.

There are no published data on toxicity or adverse effects in humans subsequent to the consumption of food products of animal origin containing traces of sulfonamides, cefaclor and fluoroquinolones. However, allergic reactions due to human exposure to sulfonamides and cefaclor are possible with mainly cutaneous symptoms ranging from a slight rash to anaphylaxis and toxic epidermal necrolysis in more severe forms [48,57]. In addition to allergic hypersensitivity reactions, quinolones might induce the emergence of antibiotic-resistant bacteria in humans, as well as organ-specific reactions in the cutaneous, hepatic and renal systems [58], hence the interest in investigating these compounds in food-producing animals.

Furthermore, from the 52 samples investigated by LC-MS/MS, 41 (78.9%) revealed the presence of 2 to 8 antibiotic residues as parent compounds and their metabolites in a single sample. This very high level could be explained by the abusive mixtures of several antimicrobials and overdosing in the treatment of various infections such as mastitis, but also by unsuitable therapeutic protocols applied by some breeders themselves in the context of self-medication (e.g., intramammary infusions) without a veterinarian prescription [14,44]. These practices have already been discussed in surveys carried out in developing countries [59], particularly as these molecules are widely accessible since they are sold over the counter at very affordable prices on the local market, as is the case in other African countries [60]. Insufficient upstream awareness and training of milk producers (e.g., lack of animal marking in the course of treatment) and collectors (who do not test for antimicrobial residues by rapid tests before each farm pass) about the presence of antibiotic residues in food of animal origin could be an important part of the problem [44]. The presence of some molecules at very high maximum concentrations, such as β-lactams and tetracyclines in the milk samples during this study, could be the consequence of farmers’ noncompliance with the withdrawal period to avoid economic losses related to the disposal of contaminated milk [4]. In Algeria, the absence of some sanction measures such as financial penalties for nonremoval of positive milk could probably lead some indelicate collectors to adopt illegal practices by delivering milk contaminated with antibiotic residues to the informal network [14]. This latter circuit, which is totally outside the scope of hygiene controls, plays an important part in the Algerian dairy sector [1]. Another possible adulteration committed by some collectors, as previously described by Orwa et al. [54], would be the addition of antibiotics for shelf-life extension of milk, particularly as these practices can be a serious hazard for consumer health.

### 3.2. Sensitivity and Specificity of the BetaStar^®^ Combo Test Compared to LC-MS/MS Results

The results of BetaStar^®^ Combo screening were compared to those of LC-MS/MS to reach conclusions about the sensitivity (true positive) and specificity (true negative) of this qualitative test. Only β-lactams and tetracyclines were included in this assessment, since the other antibiotic families such as sulfonamides and fluoroquinolones cannot be detected by the rapid screening test as stated by the manufacturer (Neogen Corporation, Lansing, MI, USA). Table 4 revealed a sensitivity of 96.2% of the BetaStar^®^ Combo for all samples, while its specificity was evaluated at 65.4%. The occurrence of false-positive in 34.6% of overall cases seems related to sample origin since it was recorded mainly for milk samples from company C (54.5%) and then company A (42.9%), while it was nil in company B. The false-negative frequency is an important feature for the evaluation of the rapid-test performance. It was lower (3.8%) than the false-compliant result required (≤5%) and found only in 1 sample as negative at rapid screening both for β-lactams and tetracycline but positive for β-lactams by LC/MS-MS from company A (14.3%). This remains in accordance with the recommendations of the European Commission [26].

The false-positive cases counted (34.6%) were less than those obtained by Ghidini et al. [18], since out of 53 positive samples to β-lactams after microbial assay screening (Delvotest^®^ SP), only 29 were confirmed by LC-MS/MS as true positives and thus 24 samples (44.4%) were false positives. However, our findings were higher than those recorded by Moat et al. [61], who reported an absence of β-lactams by HPLC for 12 samples (22.2%) from 54 presumed positives. On the contrary, under real conditions of antimicrobial residue assessment of 18 presumptive positive milk samples by BetaStar^®^ (another version than the one used in the study) and LC-ESI-MS/MS, Riedkier et al. [42] obtained only 11.1% (2 presumed false-positive cases) by the rapid-screening test. This reduced prevalence of false-positives could be explained by the double screening (Delvotest^®^ SP, BetaStar^®^) performed, which reduced the number of false noncompliants. This highlights that the number of false-positives can be fluctuating depending on the assay conditions.

**Table 4:** Sensitivity and specificity of BetaStar^®^ Combo test regarding LC-MS/MS results in all samples.

			LC-MS/MS Positivity (Total)		Total	Chi-Square Tests Value	Sig.
			Negative ^1^	Positive ^2^			
BetaStar^®^ Combo total	Negative ^1^	within LC-MS/MS	17 _a_ (65.4%)	1 _b_ (3.8%)	18 (34.6%)	21.75	0.000
	Positive ^2^	within LC-MS/MS	9 _a_ (34.6%)	25 _b_ (96.2%)	34 (65.4%)		
Total			26	26	52		
Total BetaStar^®^ Combo Sensitivity			96.2%				
Total BetaStar^®^ Combo Specificity			65.4%				
BetaStar^®^ Combo total in Company A	Negative ^1^	within LC-MS/MS	4 _a_ (57.1%)	1 _a_ (14.3%)	5 (35.7%)	2.80	0.094
	Positive ^2^	within LC-MS/MS	3 _a_ (42.9%)	6 _a_ (85.7%)	9 (64.3%)		
Total			7	7	14		
Total BetaStar^®^ Combo Sensitivity			85.7%				
Total BetaStar^®^ Combo Specificity			57.1%				
BetaStar^®^ Combo total in Company B	Negative ^1^	within LC-MS/MS	8 _a_ (100%)	0 _b_ (0.0%)	8 (57.1%)	14.00	0.000
	Positive ^2^	within LC-MS/MS	0 _a_ (0.0%)	6 _b_ (100%)	6 (42.9%)		
Total			8	6	14		
Total BetaStar^®^ Combo Sensitivity			100%				
Total BetaStar^®^ Combo Specificity			100%				
BetaStar^®^ Combo total in Company C	Negative ^1^	within LC-MS/MS	5 _a_ (45.5%)	0 _b_ (0.0%)	5 (20.8%)	7.46	0.006
	Positive ^2^	within LC-MS/MS	6 _a_ (54.5%)	13 _b_ (100%)	19 (79.2%)		
Total			11	13	24		
Total BetaStar^®^ Combo Sensitivity			100%				
Total BetaStar^®^ Combo Specificity			45.5%				

Each subscript letter denotes a subset of LC-MS/MS positivity categories whose column proportions do not differ significantly from each other at the 0.05 level using “z” test. ^1^ Negative to overall ATB family. ^2^ Positive to at least one ATB family.

**Figure 4:**
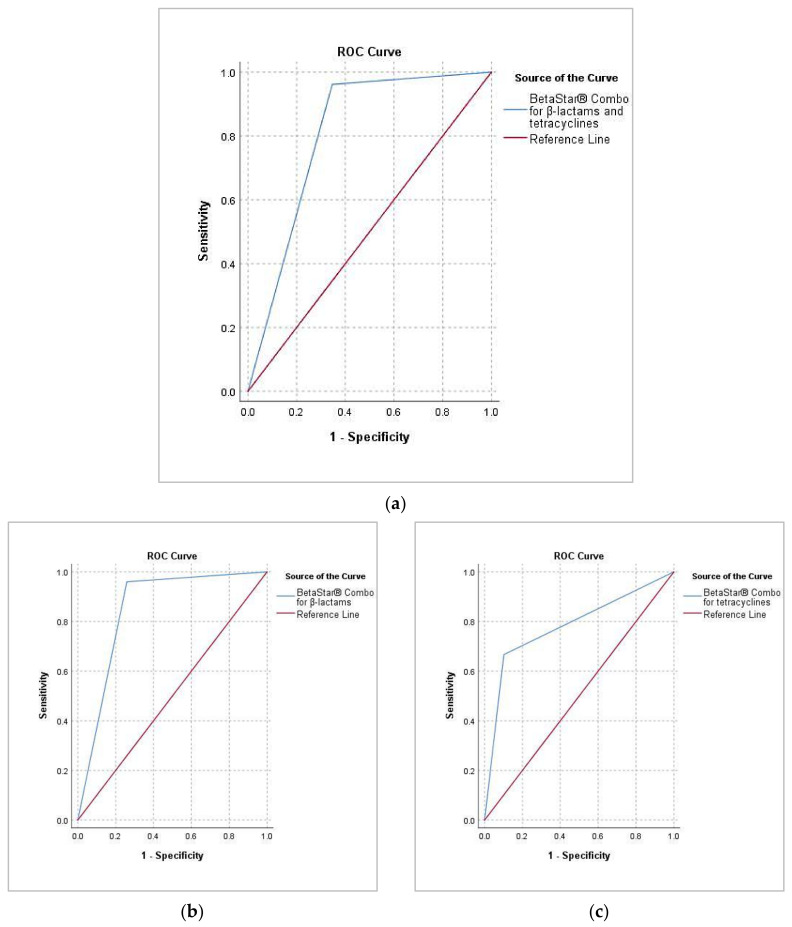
Receiver operating characteristic curve (ROC) curve obtained for the accuracy of BetaStar^®^ combo compared to LC-MS/MS for both antibiotic families (**a**) and separately, for β-lactams (**b**) and tetracyclines (**c**).

The threshold of positivity that provides the best ratio between sensitivity and specificity was defined using the Receiver Operating Characteristic (ROC) curve, which depicted the relationship between the percentage of false-positives which corresponds to 1 minus the specificity (1–Sp) and the percentage of true-positives corresponding to the sensitivity (Se). As shown in Figure 4a, the ROC curve hugs the top-left corner of the plot, which indicates that the BetaStar^®^ Combo test has good accuracy in predicting whether or not samples will get positive-to-antibiotics residues, based on their average concentrations measured by LC-MS/MS (ppb). From this plot, it appears that the Area Under the Curve (AUC) is equal to 0.81 (*p* < 0.00) and within the range of 0.8–0.9, indicating a good discriminative capacity of the BetaStar^®^ Combo test when it was applied for both families of antibiotics, β-lactams and tetracyclines, in all samples.

When compared separately by compounds family, BetaStar^®^ Combo test revealed a good discriminating capacity (range 0.8–0.9) for β-lactams with an AUC equal to 0.85 for a CI of 95% (Figure 4b) compared to tetracyclines. For the latter, the BetaStar^®^ Combo test displayed an AUC equal to 0.78 for a CI of 95% (Figure 4c), which ranged from 0.7 to 0.8, equivalent to an acceptable discriminatory capacity. Therefore, the BetaStar^®^ Combo test demonstrated a relevant performance to define the true (positive) contaminated milk samples by both groups (β-lactams and tetracyclines) nevertheless, with a better selectivity for β-lactams compared to tetracycline.

The confirmatory LC-MS/MS assessment of the 52 screened milk samples using the BetaStar^®^ Combo rapid test, however, revealed one (1) false compliant result (false-negative) and seven (7) false-positive samples to β-lactams (Table S4 in “Electronic Supplementary Material”), while only one (1) false compliant result (false-negative) and five (5) false-positive samples to tetracyclines (Table S5) were detected. Compared to false-positive, false-negative cases are scarcer; nevertheless, only 1 case was reported in 1 sample of company A, which could be explained by the low detection capability of the BetaStar^®^ Combo for cefalexin (>700 μg/kg), as reported by Reybroeck and Ooghe [23]. The EU Directive (N°37/2010) [32] established the MRLs in milk for this compound at 100 μg/kg, which can generate false-negative cases, as observed in the sample (N°35), which had a cefalexin content assessed at 111 μg/kg (see Table S6, which describes the experimental results for each milk sample involved in the LC-MS/MS analysis). With another version of BetaStar^®^ (1 + 1 protocol), Reybroeck and Ooghe [62] also recorded a poor detection capability for cefalexin (>6000 μg/kg) and for other cephalosporins (ceftiofur, cefazolin). Further suspicious cases of samples (N°16 and N°32) in companies C and A, respectively, could be explained by the occurrence at low levels of metabolites belonging to the β-lactam family, in particular metabolites of penicillin G (17.0 μg/kg) and cloxacillin (34.0 μg/kg), at concentrations that could not be detected by the BetaStar^®^ Combo rapid test due to its limited selectivity for metabolites at low concentration levels. Indeed, the literature does not report the possibility of detection of cloxacillin or penicillin G metabolites at low concentrations by using the rapid-screening test. Metabolites are classified as minor residual compounds and may still be present in milk for human consumption, but existing Algerian [12] or EU [32] regulations have only established MRLs for parent compounds. Furthermore, a false-negative sample to tetracyclines screened by the BetaStar^®^ Combo test was noticed as it was found from the LC-MS/MS analysis that it contained high oxytetracycline levels (179 μg/kg), exceeding the MRL (100 μg/kg) (sample case N°28, Table S6). One of the possible explanations for this result, according to the literature, is that of milk calcium [63]. Caseins, the main component of milk proteins, appear in the form of casein micelles that are bound together with calcium. This metallic cation has a strong ability to chelate with tetracyclines [64]. Therefore, it could interfere with the reading of the tetracycline result by BetaStar^®^ Combo and thus explain the presence of false-negative cases of oxytetracycline, thereby escaping routine control at the dairy laboratory, which may be hazardous to human health when this type of milk manages to pass unnoticed into the food chain.

Moreover, we registered cases of samples (N°33 and N°39) with positive results for the rapid-screening test, which presented high concentrations (758 and 168 μg/kg, respectively) of cloxacillin metabolites in the absence of their parent compound. Indeed, we noticed 2 false-positive samples (N°15 and 36) with parent compounds concentrations under the MRL, but with significant levels (113 and 52 μg/kg, respectively) for cloxacillin metabolites (Table S6). By using the BetaStar^®^ Combo test, Reybroeck and Ooghe [23] noted a detection capability from a concentration over 1000 μg/kg for the metabolite desfuroylceftiofur, which is 10-fold the MRL permitted for ceftiofur belonging to the β-lactam antibiotic family as cloxacillin. Beltrán et al. [24,26], while validating different commercial receptor-binding assays including the BetaStar^®^ Combo on sheep and goat milk, noted that 4-epimers (4-Epichlortetracycline, 4-Epioxytetracycline, 4-Epitetracycline), considered as tetracycline metabolites were detected at thresholds higher than the MRLs of their parent compounds. All this indicates that further work needs to be performed on the detection capability of β-lactam metabolites by BetaStar^®^ Combo. In some way, the high concentrations recorded for metabolites of penicillin G and cloxacillin in this study may explain the positivity revealed for samples subject to the rapid screening. Kits of this test are commonly calibrated on the basis of the compound parent itself, but if they are positive to metabolites, these latter may remain in the matrix for a long period of time, even after the parent compound has been removed, especially as they would constitute a potential risk assessment for consumers’ health. This demonstrates the importance of having better detection tools such as the LC method with higher precision and accuracy to detect more positive samples [65,66].

Titratable acidity represents the total concentration of acid contained in a food matrix determined by titration. It is considered the best indicator of the impact of acid content on food flavor compared to pH measurement [67].

In all three companies, the average values (Table 5) obtained were significantly different (higher or lower) from the minimum (FL: 3.5 g/100 mL; titratable acidity: 14°D) and maximum (FL: 4 g/100 mL; titratable acidity: 18°D) reference ranges estimated by Renhe et al. [68] in bovine raw milk.

Despite this significance, these values were within the reference (min and max) intervals with the exception of company C, which displayed an average titratable acidity value exceeding the maximum limit (18°D). In addition, company A (Algiers) recorded a lower mean (3.41 g/100 mL) but was not significantly different (*p* > 0.05) from the minimum reference limit (3.5 g/100 mL). The Tukey post hoc test revealed a significant difference between the means of FL recorded in companies B and C (3.15 g/100 mL vs. 3.71 g/100 mL; *p* = 0.01) and between the acidity averages recorded in the three companies (A, B and C). These intercompany differences (Table S7) confirm the effect of the samples’ origins (company) on the variation of the values of the two parameters (FL and mainly titratable acidity).

The cheese factory of Blida (company C) has certain specific features related to the activities of this company. Indeed, compared to the other industries under study, it often receives much lower daily volumes (<500 L) of milk by tanker truck from each of its affiliated collectors, due to its limited storage capacity. On the other hand, this company, unlike the others, is exclusively dedicated to the production of a single product, namely a Camembert-type cheese. This fact, combined with the weakness of the storage capacity of collected volumes, explains the tolerance of a titratable acidity close to or slightly higher than the required threshold of 18°D on the delivered milk, as we have noticed on some bulk-tank milk samples (N°16, 17, 25 and 39 in Table S6), which will be immediately directed to the cheese transformation process.

For that company, this reflects a dysfunction in the milk distribution chain between the protagonists, i.e., producers and collectors, with an abnormal lengthening of the time elapsed between milking, collection and delivery due to remoteness, leading to a rapid deterioration of milk quality such as the acceleration of acidification exacerbated with an unsuitable cooling temperature or if there is no refrigeration chain during the transportation [16,69]. In this company, compared to the others, the occurrence of some false-positive cases to the BetaStar^®^ Combo test seems to be mainly related to a higher titratable acidity level, exceeding the normal limit in tetracycline-positive milk samples compared to those (*p* = 0.001) of the negative samples (Table S8). In fact, 4 out of 5 samples classified as false-positive to this group of antibiotics were significantly prevalent (*p* < 0.05) in the class of samples exceeding the required acidity limit (18°D) and were frequently classified (*p* < 0.05) at FL values below or above the threshold range of 3.5–4 g/100 mL (Table S9). Indeed, collectors delivering lower milk quantities by tanker trucks from a few farms are more subject to fluctuations (Table 5) in FL, with minimum values as low as 2.7 g/100 mL (N°40, Table S6), which would be explained by cases of adulteration by some milk producers or collectors through the practice of skimming or by added water. In eastern and southern Africa, adulteration is a major concern for consumers, sometimes more so than the presence of antimicrobial residues [69]. In the same context, but regarding the maximum threshold value of FL, high values such as 5.6 g/100 mL or 6.2 g/100 mL (N°48 and N°30, respectively, Table S6) were recorded for BetaStar^®^ Combo false-positive cases, all belonging to the tetracycline family. Out of a total of 12 samples tested, Reybroeck and Ooghe [23] reported 1 case of a false-positive sample for β-lactams and another one for tetracyclines on milk with low fat content (<2 g/100 mL) when screened by BetaStar^®^ Combo. These authors also reported false-positive cases correlated with a high fat content (>6 g/100 mL), especially for the tetracycline family, which is in accordance with our findings. The occurrence of false-positive cases by the BetaStar^®^ Combo test could be explained by a slow flow rate in the tank, favored by high fat content. These findings are in agreement with those of Nouws et al. [70], who reported false-positives for tetracycline residues in bulk milk using another test (Charm HVS); this would be correlated with the fatty-acid content present. Conversely, Mirecki and Nikolić [19] mentioned that FL content in the range of 1.8–5.6% did not affect the detection limit of oxytetracycline with the Delvotest^®^ Accelerator.

Table 5 also shows a significant (*p* < 0.05) influence of the company on the variation of milk fat content. The occurrence of higher fat levels (3.71 ± 0.73 g/100 mL) in company C would be related to the seasonal effect of sampling, coinciding with the availability of a ration rich in fodder, in concentrates and the grazing practice in the Mitidja’s plains of Blida region during the spring [71].

In addition to the large fluctuations in fat content in company C, we also noticed quite marked titratable acidity values in several samples from the collection or from the tank of the cheese dairy. Indeed, a high value (≥19°D) of titratable acidity of the milk, whether or not coupled to higher or lower FLs values, could explain the occurrence of false-positive cases, more particularly those belonging to the tetracycline family, as we have noticed for samples N°25, 30, 48, 52 (Table S6). Reybroeck and Ooghe [23] reported that when acidic milk displayed a low pH (6.0) value on blank milk and milk fortified with Benzylpenicillin, false-positive cases to tetracyclines were observed. In addition, we also identified further cases (samples N°47 and 50) of false-positives belonging to β-lactams, on milk with a titratable acidity ranging from 18.5 to 20°D, thus above the interval requested for raw milk (Table S6). Furthermore, it is worth highlighting the case of sample N°49 belonging to company A, detected as false-positive by the BetaStar^®^ Combo, which revealed a normal value (3.1 g/100 mL) for FLs, as well as for titratable acidity (15°D). This could be explained either by abnormally low or high levels of milk protein, or by elevated levels of somatic cells (>10^6^ per mL), especially because of the high frequency of clinical and subclinical mastitis recorded in several Algerian farms affecting the hygienic quality of the collected milk [2,16]. In agreement with what we previously supposed, Grooms et al. [72] noticed that the presence of high protein, FL and somatic cell count (SCC) levels, especially at the end of the lactation period, increased the probability of false-positive results for ceftiofur residues in milk samples screened by another version of the test, BetaStar^®^ Plus assay.

## 4. Conclusions

The present study revealed the importance of quality control of collected raw milk, as there is a conspicuous number of false positives for β-lactam and tetracycline residues after using the rapid screening test. Moreover, the efficiency of LC-MS/MS as a confirmatory tool in the accuracy assessment of these tests is established. Milk quality control should take into account the acidity and fat content of the milk that could cause a false decision leading to rejection of compliant products. This work also demonstrated that the rapid-screening test was able to detect in the field conditions a significant number of molecules belonging to β-lactams and tetracyclines at levels close to the MRLs, as confirmed by LC-MS/MS analysis. However, substances not detected by this rapid test and therefore not currently investigated by the studied companies, such as fluoroquinolones, sulfonamides and cefaclor, require particular attention as they may go undetected at the time of control and may expose the consumers to potential health risks. Given the unavailability of official data, the very high concentrations found for certain families of antibiotics also reinforced the importance of implementing a national control program for the detection of drug residues in food matrices of animal origin. For this purpose, the LC-MS/MS method could be effective in strengthening the control points. For future research, it would be important to monitor the metabolites of β-lactams and tetracyclines, and to check at what limits these could be detected by rapid-screening tests. Finally, it is crucial to involve milk producers, veterinary practitioners and collectors in a national plan to assess the ecotoxicological hazard of drug use in Algerian livestock.

## Figures and Tables

**Table 1:** Limits of detection for antimicrobial drugs by BetaStar^®^ Combo and LC-MS/MS.

Group	Substance	MRL (µg/kg) *	Limit of Detection Beta Star Combo (µg/kg) **	Limit of Detection LC-MS/MS (µg/kg)
Penicillins	Penicillin V	–	–	1
	Benzylpenicillin	4	4	1
	Ampicillin	4	4	1
	Amoxicillin	4	4	1
	Oxacillin	30	5	1
	Cloxacillin	30	5	1
	Dicloxacillin	30	6	1.5
	Nafcillin	30	12	–
Cefalosporines	Ceftiofur	100	90	15
	Desfuroylceftiofur	100	1000	–
	Cefquinome	20	8	3.5
	Cefazolin	50	40	2.5
	Cephapirin	60	9	5
	Desacetylcephapirin	60	3	–
	Cefacetrile	125	40	–
	Cefoperazone	50	8	5
	Cefalexin	100	700	2
	Cefalonium	20	5	2
Tetracyclines	Tetracycline	100	100	1.7
	Oxytetracycline	100	100	1.7
	Chlortetracycline	100	35	1.7
	Doxycycline	–	14	1.7

* European Regulatory Commission 2010/37/EC [32]. ** Reybroeck and Ooghe [23].

**Table 2:** Milk samples’ proportions tested positive or negative for antibiotic residues (case number) by BetaStar^®^ Combo.

Algerian Dairy Company				Company A (Algiers)	Company B (Boumerdes)	Company C (Blida)	Total
Samples screened by BetaStar^®^ Combo	Total			186	140	119	445
	Positive	Total		9 (4.84%)	6 (4.29%)	19 (16.0%)	34 (7.64%)
		to β-lactams (Sum)		9 (100%)	6 (100%)	16 * (84.2%)	31 * (91.2%)
		Only to β-lactams		9 (100%)	6 (100%)	12 (63.2%)	27 (79.4%)
		to tetracyclines (Sum)		-	-	7 * (36.8%)	7 * (20.6%)
		Only to tetracyclines		-	-	3 (15.8%)	3 (8.82%)
		to β-lactams and tetracyclines		-	-	4 (21.1%)	4 (11.8%)
	Negative			177 (95.2%)	134 (95.7%)	100 (84.0%)	411 (92.4%)
Samples screened by BetaStar^®^ Combo and assessed by LC-MS/MS	Total			14	14	24	52
	Positive **			9 (64.3%)	6 (42.9%)	19 (79.2%)	34 (65.4%)
	Negative ***			5 (35.7%)	8 (57.1%)	5 (20.8%)	18 (34.6%)
Samples screened by LC-MS/MS	Residue presence		Total	14 (100%)	12 (85.7%)	21 (87.5%)	47 (90.4%)
			Positive (>MRL)	7 (50.0%)	6 (50.0%)	13 (61.9%)	26 (55.3%)
			Negative (≤MRL)	7 (50.0%)	6 (50.0%)	8 (38.1%)	21 (44.7%)
	Residues absence			0 (0%)	2 (14.3%)	3 (12.5%)	5 (9.62%)
	Total screened			14 (100%)	14 (100%)	24 (100%)	52 (100%)

* A sample can be positive for both families’ β-lactams and tetracyclines. ** All BetaStar^®^ Combo positive samples were assessed by LC-MS/MS. *** Negative bulk-tank milk samples to BetaStar^®^ Combo were assessed by LC-MS/MS.

**Table 3:** Frequency of antimicrobial residues in milk assessed by LC-MS/MS in the studied dairy companies.

ATB Family	Compounds	Total Compounds in All Samples				MRL µg/kg [12,32]	Negative (≤MRL)			Positive (>MRL)		
		n	% ^a^	% ^b^	Min–Max µg/kg		*n*	% ^a^	% ^b^	*n*	%^a^	% ^b^
β-lactams	Ampicillin *	6	3.73	11.5	6.1–309	4	0	0.00	0.00	6	8.82	11.5
	Cefalexin *	2	1.24	3.85	1.4–111	100	1	1.11	1.92	1	1.47	1.92
	Cefazolin	1	0.62	1.92	50	50	1	1.11	1.92	0	0.00	0.00
	Cefoperazone	1	0.62	1.92	17	50	1	1.11	1.92	0	0.00	0.00
	Cloxacillin (M) *	26	16.2	50.0	4.9–1505	-						
	Cloxacillin (P) *	16	9.94	30.8	3.9–1231	30	5	5.56	9.62	11	16.2	21.2
	Dicloxacillin (M) *	6	3.73	11.5	1.0–893	-						
	Dicloxacillin (P) *	2	1.24	3.85	1.8–413	30	1	1.11	1.92	1	1.47	1.92
	Oxacillin (M)	3	1.86	5.77	0.49–1.1	-						
	Oxacillin (P) *	2	1.24	3.85	18–36	30	1	1.11	1.92	1	1.47	1.92
	Penicillin G (M) *	17	10.6	32.7	4.0–2115	-						
	Penicillin G (P) *	10	6.21	19.2	28–2062	4	0	0.00	0.00	10	14.7	19.2
	Cefaclor **	3	1.86	5.8	81–220	-						
	Total	95	59.0				10	11.1		30	44.1	
Tetracycline	Chlortetracycline	2	1.24	3.85	7.9–12	100	2	2.22	3.85	0	0.00	0.00
	Oxytetracycline *	18	11.2	34.6	5.9–660	100	16	17.8	30.8	2	2.94	3.85
	Tetracycline *	2	1.24	3.85	40–2291	100	1	1.11	1.92	1	1.47	1.92
	Total	22	13.7				19	21.1		3	4.41	
Fluoroquinolones	Ciprofloxacin	4	2.48	7.69	3.2–33	100 ^c^	4	4.44	7.69	0	0.00	0.00
	Danofloxacin	1	0.62	1.92	8.5	30	1	1.11	1.92	0	0.00	0.00
	Enrofloxacin	8	4.97	15.4	1.5–100	100 ^c^	8	8.89	15.4	0	0.00	0.00
	Flumequine *	22	13.7	42.3	0.27–52	50	21	23.3	40.4	1	1.47	1.92
	Marbofloxacin	2	1.24	3.85	0.89–51	75	2	2.22	3.85	0	0.00	0.00
	Total	37	23.0				36	40.0		1	1.47	
Sulfonamide	Sulfachloropyridazine	1	0.62	1.92	5.70	100 ^d^	1	1.11	1.92	0	0.00	0.00
	Sulfadimethoxine	1	0.62	1.92	6.90	100 ^d^	1	1.11	1.92	0	0.00	0.00
	Sulfadimidine	1	0.62	1.92	58.0	25 [12]	0	0	0	1	1.11	1.92
	Sulfamerazine	1	0.62	1.92	0.82	100 ^d^	1	1.11	1.92	0	0.00	0.00
	Sulfapyridine	1	0.62	1.92	3.60	100 ^d^	1	1.11	1.92	0	0.00	0.00
	Sulfathiazole	1	0.62	1.92	5.10	100 ^d^	1	1.11	1.92	0	0.00	0.00
	Total	6	3.73				6	6.67				
Trimethoprim (Diaminopyrimidines)		1	0.62	1.92	16.0	50	1	1.11	1.92	0	0.00	0.00
Total		161	100 ^a^				72	100 ^a^		34	100 ^a^	

* Positive compound. ** Parental compound without MRL and not prescribed in dairy cows. n compounds frequency. ^a^ Percent per total compound (161) found in all samples (52) assessed by LC-MS/MS. ^b^ Percent per total samples (52) assessed by LC-MS/MS. ^c^ Sum of enrofloxacin and ciprofloxacin should not exceed 100 µg/kg. ^d^ The combined total residues of all compounds within the sulfonamide group should not exceed 100 µg/kg. MRL not established.

**Table 5:** Analysis of FL and titratable acidity variances across companies.

Parameters			FL (g/100 mL)				Titratable Acidity (°D)			
Companies			Company A	Company B	Company C	Total	Company A	Company B	Company C	Total
N of samples			14	14	24	52	14	14	24	52
Mean			3.41 ^b^	3.15 ^a,b^	3.71 ^a,b^	3.48	15.1 ^a,b^	16.4 ^a,b^	18.9 ^a,b^	17.2
Std. Deviation			0.38	0.17	0.73	0.59	1.04	1.22	1.27	2.04
95% CI for Mean	LowerBound		3.20	3.05	3.40	3.32	14.5	15.7	18.4	16.7
	UpperBound		3.63	3.25	4.02	3.64	15.7	17.1	19.5	17.8
Minimum			3.00	2.80	2.70	2.70	13.00	15.0	17.0	13.0
Maximum			4.20	3.50	6.20	6.20	16.50	18.0	23.0	23.0
Reference range ^1^: Min–Max			3.5–4	3.5–4	3.5–4	3.5–4	14–18	14–18	14–18	14–18
Sig. of Tukey’s HSD test		Company A		0.41	0.25			0.01 *	0.00 *	
		Company B	0.41		0.01 *		0.01 *		0.00 *	
		Company C	0.25	0.01 *			0.00 *	0.00 *		

* The mean difference between companies is significant at the 0.05 level using Tukey’s HSD test for multiples comparison. ^a^ significant difference of the mean with minimum reference value (*p* < 0.05). ^b^ significant difference of the mean with maximum reference value (*p* < 0.05). ^1^ Renhe et al. [68].

## Data Availability

All data are reported in the tables and figures of the manuscript.

## Supplementary Materials

The following are available online at https://www.mdpi.com/article/10.3390/toxics10010019/s1, Table S1: Validation data for β-lactams determination using LC-MS/MS, Table S2: Gradient elution programs for the determination of antibiotic residues, Table S3: MRM parameters and retention times for all compounds determined, Table S4: Sensitivity and specificity of BetaStar^®^ Combo test regarding to LC-MS/MS for β-lactams, Table S5: Sensitivity and specificity of BetaStar^®^ Combo test regarding to LC-MS/MS for tetracycline, Table S6: Experimental results by individual code for each milk sample analyzed by LC-MS/MS, Table S7: Relationship between variables (FL, acidity, and samples origin) and the results of screening using BetaStar^®^ Combo and LC-MS/MS, Table S8: Comparison of FL (g/100 mL) and titratable acidity (°D) means following the BetaStar^®^ Combo test result, Table S9: Variance of fat levels and acidity according to the accuracy of the rapid screening test for ß-lactams and tetracyclines.
