# Supplementary material for: Comparative Assessment of Antibiotic Residues Using Liquid Chromatography Coupled with Tandem Mass Spectrometry (LC-MS/MS) and a Rapid Screening Test in Raw Milk Collected from the North-Central Algerian Dairies
Source: Toxics. 2022 Jan 5;10(1):19. doi: 10.3390/toxics10010019 (PMC8781432; doi:10.3390/toxics10010019)
Supplement: Supplementary file 1 [file toxics-10-00019-s001.zip › toxics-1498413-sm-final/toxics-1498413 -sm-final.pdf]

toxics

MDPI

Supplementary Materials: Comparative Assessment of Antibiotic Residues Using Liquid Chromatography Coupled with Tandem Mass Spectrometry (LC-MS/MS) and a Rapid Screening Test in Raw Milk Collected from the North-Central Algerian Dairies

Fawzi Rostane Meklati, Anthi Panara, Ahmed Hadef, Amel Meribai, Meriem H. Ben-Mahdi, Marilena E. Dasenaki and Nikolaos S. Thomaidis

Table S1. Validation data for β-lactams determination using LC-MS/MS.

Compound	Linearity*	% Recoveries**	%CV$_{Reproducibility}$ (n = 10)
Cefaclor	$y = 780.36x{-}5938.2$, $R^2 = 0.994$	89.2	12
Cefadroxil	$y = 819.11x{-}9585.6$, $R^2 = 0.98$	94.0	8.9
Cefazolin	$y = 2924.6x{-}50031$, $R^2 = 0.990$	86.9	14
Cefalexin	$y = 6450.9x{-}69248$, $R^2 = 0.993$	91.1	9.3
Cefquinome	$y = 9874.6x{-}222158$, $R^2 = 0.98$	90.3	10
Cefalonium	$y = 2671.2x{-}31728$, $R^2 = 0.990$	98.0	11
Ceftiofur	$y = 829.1x{-}18653$, $R^2 = 0.98$	96.1	11
Cefapirin	$y = 14117x{-}129611$, $R^2 = 0.995$	89.0	15
Cefoperazone	$y = 172.15x{-}2423$, $R^2 = 0.992$	96.4	14
Amoxicillin	$y = 650.2x{-}3322.4$, $R^2 = 0.992$	88.8	7.9
Ampicillin	$y = 8715.4x{-}118331$, $R^2 = 0.995$	80.4	12
Cloxacillin	$y = 16202x{-}355295$, $R^2 = 0.990$	86.7	16
Oxacillin	$y = 9022.3x{-}197855$, $R^2 = 0.990$	95.5	14
Dicloxacillin	$y = 10217x{-}231962$, $R^2 = 0.98$	92.7	11
Penicillin V	$y = 13008x{-}3441.2.$, $R^2 = 0.992$	80.4	10
Penicillin G	$y = 777.81x{-}10561$, $R^2 = 0.995$	97.3	10

*std addition calibration curve : 15–400 μg/kg. **C spike for recovery calculation = 100 μg/kg.

Table S2. Gradient elution programs for the determination of antibiotic residues.

GRADIENT PROGRAMME **sulfonamides, tetracyclines, quinolones, macrolides and trimethoprim**		*GRADIENT PROGRAMME* **β-lactams**	
Time (min)	% B	Time (min)	% B
0	10	0	5
10	100	5	70
27	100	9	100
		13	100

Table S3. MRM parameters, retention times and ion ratio for all compounds determined.

Compound	Pseudo Molecular Ion	Product Ion 1	Collision Energy (eV)	Product Ion 2	Collision Energy (eV)	Tube Lens	RT (min)	Ion Ratio %
Quinolones								
Ciprofloxacin	332	**288**	18	314	22	85	7.7	41
Danofloxacin	358	**96**	25	314	20	85	7.9	14
Difloxacin	400	**356**	20	299	27	85	8.0	75
Enrofloxacin	360	**316**	25	342	20	85	7.7	91
Flumequine	262	**244**	20	202	30	85	12.0	37
Marbofloxacin	363	**320**	15	72	20	85	7.2	51
Norfloxacin	320	**276**	16	233	23	91	7.6	72
Ofloxacin	362	**318**	19	261	27	120	7.4	93
Oxolinic acid	262	**244**	18	158	31	79	10.7	2.5
Sarafloxacin	386	**342**	18	299	27	85	8.1	95
Tetracyclines								
Chlortetracycline	479	**444**	20	462	15	90	9.4	58
Doxycycline	445	**427**	19	267	35	90	10.1	25
Oxytetracycline	461	**426**	19	443	12	90	8.4	24
Tetracycline	445	**410**	18	426	12	90	8.2	8.0
Cefalosporines								
Cefaclor	368	**174**	14	106	32	81	7.7	58
Cefadroxil	364	**346**	19	114	29	97	6.6	30
Cefazolin	455	**156**	11	323	17	86	8.3	80
Cefalexin	348	**174**	6	158	30	113	7.6	72
Cefquinome	529	**134**	16	396	11	83	6.9	82
Cefalonium	459	**337**	9	152	22	83	7.7	50
Ceftiofur	524	**241**	25	210	31	97	9.4	65
Cefapirin	424	**292**	14	152	23	85	6.8	85
Cefoperazone	646	**143**	32	529	12	90	8.4	80
Penicillins								
Amoxicillin	366	**349**	8	114	22	68	6.6	67
Ampicillin	350	**106**	20	160	12	87	7.9	66
Cloxacillin	436	**114**	33	276.7	13	84	11.5	30
Oxacillin	402	**243**	13	114	32	65	12.0	35
Dicloxacillin	470	**311**	15	114	33	101	12.0	36
Penicillin V	351	**160**	11	114	31	64	11.3	60
Penicillin G	335	**176**	15	160	10	87	10.7	40
metabolite of Cloxacillin	410	**178**	32	174	19	80	11.0	60
metabolite of Oxacillin	376	**174**	18	144	31	97	10.6	96
metabolite of Penicillin V	325	**128**	26	174	16	86	10.5	79
metabolite of Dicloxacillin	444	**128**	33	211	29	115	11.4	40
metabolite of Penicillin G	309	**174**	16	**128**	26	93	9.9	79
Macrolides								
Azithromycin	750	**158**	29	591	37	127	8.4	48
Clarithromycin	749	**158**	30	590	20	123	11.1	41
Erythromycin	734	**158**	30	576	20	130	10.4	75
Tiamullin	494	**192**	21	119	33	101	10.2	28
Tilmicosin	869	**174**	42	156	44	165	8.9	38
Tylosin	917	**174**	36	772	28	148	10.3	36

				Sulfonamides				
Sulfaclozine	285	**156**	28	92	15	87	10.1	47
Sulfachloropyridazine	285	**156**	28	92	14	87	9.0	28
Sulfadimidine	279	**186**	17	124	26	87	8.8	34
Sulfadimethoxine	311	**156**	17	108	29	87	10.1	43
Sulfadoxine	311	**156**	17	108	27	87	9.2	32
Sulfadiazine	251	**156**	15	92	27	87	7.4	55
Sulfaguanidine	215	**156**	14	92	14	87	4.2	2.0
Sulfisoxazole	268	**156**	13	92	27	87	8.2	65
Sulfamonomethoxine	281	**92**	13	156	29	87	8.6	90
Sulfamethoxypyridazine	281	**156**	13	92	29	87	9.2	90
Sulfamerazine	265	**172**	16	156	16	87	8.1	98
Sulfamethizole	271	**156**	14	92	28	87	8.3	38
Sulfamethoxazole	254	**156**	16	108	25	87	9.0	60
Sulfamoxole	268	**156**	13	92	28	87	9.2	44
Sulfapyridine	250	**156**	15	184	17	87	7.6	70
Sulfaquinoxaline	301	**156**	18	92	30	87	10.4	38
Sulfathiazole	256	**156**	15	92	26	87	7.4	39
				Other antibiotics				
Trimethoprim	291	**230**	25	123	30	87	7.1	18

Table S4. Sensitivity and specificity of BetaStar® Combo test regarding to LC-MS/MS for β-lactams.

			**LC-MS/MS positivity to β-lactams**		**Total**	**Chi-Square Tests Value**	**Sig.**
			**Negative**	**Positive**			
BetaStar® Combo for β-lactams (all samples)	Negative	within LC-MS/MS	20_a (74.1%)	1_b (4%)	21 (40.4%)	26.475	0.000
	Positive	within LC-MS/MS	7_a (25.9%)	24_b (96%)	31 (59.6%)		
Total			27	25	52		
Total BetaStar® Combo Sensitivity					96%		
Total BetaStar® Combo Specificity					74.1%		
BetaStar® Combo for β-lactams in Company A	Negative	within LC-MS/MS	4_a (57.1%)	1_a (14.3%)	5 (35.7%)	2.800	0.094
	Positive	within LC-MS/MS	3_a (42.9%)	6_a (85.7%)	9 (64.3%)		
Total			7	7	14		
Total BetaStar® Combo Sensitivity					85.7%		
Total BetaStar® Combo Specificity					57.1%		
BetaStar® Combo for β-lactams in Company B	Negative	within LC-MS/MS	8_a (100%)	0_b (0.0%)	8 (57.1%)	14.000	0.000
	Positive	% within LC-MS/MS	0_a (0.0%)	6_b (100%)	6 (42.9%)		
Total			8	6	14		
Total BetaStar® Combo Sensitivity					100%		
Total BetaStar® Combo Specificity					100%		
BetaStar® Combo for β-Lactams in Company C	Negative	% within LC-MS/MS	8_a (66.7%)	0_b (0.0%)	8 (33.3%)	12.000	0.001
	Positive	% within LC-MS/MS	4_a (33.3%)	12_b (100%)	16 (66.7%)		
Total			12	12	24		
Total BetaStar® Combo Sensitivity					100%		
Total BetaStar® Combo Specificity					66.7%		

Each subscript letter denotes a subset of LC-MS/MS positivity categories whose column proportions do not differ significantly from each other at the 0.05 level using "z" test.

Table S5. Sensitivity and specificity of BetaStar® Combo test regarding to LC-MS/MS for tetracycline.

			LC-MS/MS positivity to tetracycline		Total	Chi-Square Tests Value	Sig.
			Negative	Positive			
BetaStar® Combo for tetracycline (all samples)	Negative	within LC-MS/MS	44_a (89.8%)	1_b (33.3%)	45 (86.5%)	7.736	0.005
	Positive	within LC-MS/MS	5_a (10.2%)	2_b (66.7%)	7 (13.5%)		
Total			49	3	52		
Total BetaStar® Combo Sensitivity					66.7%		
Total BetaStar® Combo Specificity					89.8%		
BetaStar® Combo for tetracycline in Company A	Negative	within LC-MS/MS	14 (100%)	0 /	14 (100%)	Ns	
	Positive	within LC-MS/MS	0	0	0		
Total			14	0	14		
Total BetaStar® Combo Sensitivity							
Total BetaStar® Combo Specificity					100%		
BetaStar® Combo for tetracycline in Company B	Negative	within LC-MS/MS	14 (100%)	0 /	14 (100%)	Ns	
	Positive	within LC-MS/MS	0	0	0		
Total			14	0	14		
Total BetaStar® Combo Sensitivity					/		
Total BetaStar® Combo Specificity					100%		
BetaStar® Combo for tetracycline in Company C	Negative	within LC-MS/MS	16_a (76.2%)	1_a (33.3%)	17 (70.8%)	2.334	0.127
	Positive	within LC-MS/MS	5_a (23.8%)	2_a (66.7%)	7 (29.2%)		
Total		Count	21	3	24		
Total BetaStar® Combo Sensitivity					66.7%		
Total BetaStar® Combo Specificity					76.2%		

Each subscript letter denotes a subset of LC-MS/MS positivity categories whose column proportions do not differ significantly from each other at the 0.05 level using "z" test. Ns. No statistics are computed because BetaStar® Combo and LC-MS/MS for tetracycline are constants.

Table S7. Relationship between variables (FL, acidity, and samples origin) and the results of screening using BetaStar® Combo and LC-MS/MS.

Variables	FL			Acidity			Companies		
	N	Pearson Correlation	Sig.	N	Pearson Correlation	Sig.	N	Pearson Correlation	Sig.
FL	52	1		52	0.31*	0.03	52	0.26	0.07
Acidity	52	0.31*	0.03	52	1		52	0.81**	0.00
Companies	52	0.26	0.06	52	0.81**	0.00	52	1	
BetaStar® Combo for all samples	52	0.18	0.21	52	0.21	0.14	52	0.17	0.23
BetaStar® Combo for β-lactams	52	−0.20	0.16	52	0.06	0.67	52	0.05	0.73
BetaStar® Combo for tetracycline	52	0.50**	0.00	52	0.45**	0.00	52	0.38**	0.005
LC-MS/MS for all samples	52	−0.13	0.37	52	0.05	0.71	52	0.05	0.75
LC-MS/MS for β-lactams	52	−0.17	0.24	52	0.02	0.90	52	0.01	0.95
LC-MS/MS for tetracycline	52	0.16	0.24	52	0.18	0.21	52	0.24	0.09
LC-MS/MS for other antibiotics	52	−0.03	0.85	52	−0.22	0.12	52	−0.17	0.24

*. Correlation is significant at the 0.05 level. **. Correlation is significant at the 0.01 level.

Table S8. Comparison of FL (g/100 mL) and titratable acidity (°D) means following the BetaStar® Combo test result.

BetaStar® Combo	Parameters	Results	N	Mean	S.D	t	Sig.
Screening for β-lactams	FL	Negative	21	3.62	0.83	1.22	0.23
		Positive	31	3.39	0.33		
	Acidity	Negative	21	17.07	1.87	−0.43	0.67
		Positive	31	17.32	2.17		
Screening for tetracycline	FL	Negative	45	3.37	0.30	−1.79	0.12
		Positive	7	4.21	1.25		
	Acidity	Negative	45	16.87	1.84	−3.52	0.00*
		Positive	7	19.50	1.80		

Table S9. Variance of fat levels and acidity according to the accuracy of the rapid screening test for ß-lactams and tetracyclines.

		BetaStar® Combo for ß-lactams				BetaStar® Combo for tetracyclines			
Positivity groups		False negative	True negative	False positive	True positive	False negative	True negative	False positive	True positive
Frequency		1	20	7	24	1	44	5	2
Percent (%)		1.92	38.46	13.46	46.15	1.92	84.62	9.62	3.85
Fat Level (g/100 mL)	Mean	3.20	3.64	3.39	3.39	3.60	3.36	4.30	4.00
	SD		0.84	0.24	0.35		0.30	1.51	0.14
	Min	3.20	3.00	3.00	2.70	3.60	2.80	2.70	3.90
	Max	3.20	6.20	3.60	4.20	3.60	4.20	6.20	4.10
	F	0.82				5.59			
	Sig.	0.49				0.002			
	FL < 3.5	1 (3.45%)$_{a}$	11 (37.93%)$_{a.\,b}$	3 (10.34%)$_{a}$	14 (48.28%)$_{a}$	0 (0%)$_{a}$	27 (93.10%)$_{a}$	2 (6.90%)$_{a}$	0 (0%)$_{a}$
	FL 3.5–4	0 (0%)$_{a}$	5 (27.78%)$_{b}$	4 (22.22%)$_{a}$	9 (50.00%)$_{a}$	1 (5.56%)$_{a}$	15 (83.33%)$_{a.\,b}$	1 (5.56%)$_{a}$	1 (5.56%)$_{a.\,b}$
	FL > 4	0 (0%)$_{a}$	4 (80.00%)$_{a}$	0 (0%)$_{a}$	1 (20.00%)$_{a}$	0 (0%)$_{a}$	2 (40.00%)$_{b}$	2 (40.00%)$_{b}$	1 (20.00%)$_{b}$
	χ2 Value	6.31				13.29			
	Asymp. Sig.	0.39				0.04			
TitratableAcidity (D°)	Mean	16.00	17.13	17.36	17.31	19.00	16.82	19.90	18.50
	SD		1.90	2.25	2.19		1.84	2.01	0.71
	Min	16.00	14.00	15.00	13.00	19.00	13.00	17.50	18.00
	Max	16.00	20.00	21.00	23.00	19.00	21.00	23.00	19.00
	F	0.15				4.89			
	Sig.	0.93				0.005			
	Acidity < 14	0 (0%)$_{a}$	0 (0%)$_{a}$	0 (0%)$_{a}$	1 (100%)$_{a}$	0 (0%)$_{a}$	1 (100%)$_{a.\,b}$	0 (0%)$_{a.\,b}$	0 (0%)$_{a}$
	Acidity 14–18	1 (2.78%)$_{a}$	15 (41.67%)$_{a}$	4 (11.11%)$_{a}$	16 (44.44%)$_{a}$	0 (0%)$_{a}$	34 (94.44%)$_{b}$	1 (2.78%)$_{b}$	1 (2.78%)$_{a}$
	Acidity > 18	0 (0%)$_{a}$	5 (33.33%)$_{a}$	3 (20.00%)$_{a}$	7 (46.67%)$_{a}$	1 (6.67%)$_{a}$	9 (60.00%)$_{a}$	4 (26.67%)$_{a}$	1 (6.67%)$_{a}$
	χ2Value	2.44				10.82			
	Asymp. Sig.	0.88				0.09			

Each subscript letter denotes a subset of parameters class categories whose column proportions do not differ significantly from each other at the 0.05 level using z test.
